# Post-radiotherapy stage III/IV non-small cell lung cancer radiomics research: a systematic review and comparison of CLEAR and RQS frameworks

**DOI:** 10.1007/s00330-024-10736-1

**Published:** 2024-04-16

**Authors:** Kevin Tran, Daniel Ginzburg, Wei Hong, Ulrike Attenberger, Hyun Soo Ko

**Affiliations:** 1 Department of Cancer Imaging, Peter MacCallum Cancer Centre, 305 Grattan St, Melbourne, VIC 3000 Australia; 2 Faculty of Medicine, Dentistry & Health Sciences, University of Melbourne, Parkville, VIC 3052 Australia; 3 Department of Diagnostic and Interventional Radiology, Venusberg Campus 1, 53127 Bonn, Germany; 4 Personalised Oncology Division, The Walter and Eliza Hall Institute of Medical Research, Melbourne, VIC Australia; 5 The Sir Peter MacCallum Department of Oncology, University of Melbourne, 305 Grattan St, Melbourne, VIC 3000 Australia

**Keywords:** Radiomics, Lung neoplasms, Radiotherapy, Multidetector computed tomography, Methods

## Abstract

**Background:**

Lung cancer, the second most common cancer, presents persistently dismal prognoses. Radiomics, a promising field, aims to provide novel imaging biomarkers to improve outcomes. However, clinical translation faces reproducibility challenges, despite efforts to address them with quality scoring tools.

**Objective:**

This study had two objectives: 1) identify radiomics biomarkers in post-radiotherapy stage III/IV nonsmall cell lung cancer (NSCLC) patients, 2) evaluate research quality using the CLEAR (CheckList_for_EvaluAtion_of_Radiomics_research), RQS (Radiomics_Quality_Score) frameworks, and formulate an amalgamated CLEAR-RQS tool to enhance scientific rigor.

**Materials and methods:**

A systematic literature review (Jun-Aug 2023, MEDLINE/PubMed/SCOPUS) was conducted concerning stage III/IV NSCLC, radiotherapy, and radiomic features (RF). Extracted data included study design particulars, such as sample size, radiotherapy/CT technique, selected RFs, and endpoints. CLEAR and RQS were merged into a CLEAR-RQS checklist. Three readers appraised articles utilizing CLEAR, RQS, and CLEAR-RQS metrics.

**Results:**

Out of 871 articles, 11 met the inclusion/exclusion criteria. The Median cohort size was 91 (range: 10–337) with 9 studies being single-center. No common RF were identified. The merged CLEAR-RQS checklist comprised 61 items. Most unreported items were within CLEAR’s “methods” and “open-source,” and within RQS’s “phantom-calibration,” “registry-enrolled prospective-trial-design,” and “cost-effective-analysis” sections. No study scored above 50% on RQS. Median CLEAR scores were 55.74% (32.33/58 points), and for RQS, 17.59% (6.3/36 points). CLEAR-RQS article ranking fell between CLEAR and RQS and aligned with CLEAR.

**Conclusion:**

Radiomics research in post-radiotherapy stage III/IV NSCLC exhibits variability and frequently low-quality reporting. The formulated CLEAR-RQS checklist may facilitate education and holds promise for enhancing radiomics research quality.

**Clinical relevance statement:**

Current radiomics research in the field of stage III/IV postradiotherapy NSCLC is heterogenous, lacking reproducibility, with no identified imaging biomarker. Radiomics research quality assessment tools may enhance scientific rigor and thereby facilitate radiomics translation into clinical practice.

**Key Points:**

- *There is heterogenous and low radiomics research quality in postradiotherapy stage III/IV nonsmall cell lung cancer.*
- *Barriers to reproducibility are small cohort size, nonvalidated studies, missing technical parameters, and lack of data, code, and model sharing.*
- *CLEAR (CheckList_for_EvaluAtion_of_Radiomics_research), RQS (Radiomics_Quality_Score), and the amalgamated CLEAR-RQS tool are useful frameworks for assessing radiomics research quality and may provide a valuable resource for educational purposes in the field of radiomics.*

## Introduction

Lung cancer is the second most prevalent malignancy worldwide, with approximately 2.2 million newly diagnosed cases in 2020 [1], the majority of which are nonsmall cell lung cancer (NSCLC), comprising nearly 84% of cases [2]. NSCLC stage I and II are typically surgically managed, while treatment for locally advanced unresectable stage III and metastatic stage IV often necessitates adjuvant radiotherapy, frequently combined with chemotherapy and sometimes immunotherapy.

Despite therapeutic advancements, there has been only marginal improvement in the 5-year survival rates for stage III/IV from 24.6% in 2016 to 26.4% in 2020 [2]. Consequently, the research focus has shifted towards screening, diagnosis, and personalized management strategies to ameliorate both quality of life and survival outcomes.

Radiomics, an emerging field, leverages noninvasive techniques to extract radiomic features (RFs) from medical images, surpassing standard radiology reporting. RFs, also known as texture analysis, capture grey-level intensities and spatial relationships within the region of interest (ROI) in two-dimensional (2D) pixel and three-dimensional (3D) voxel spaces, hypothesized to be associated with tissue heterogeneity and tumor microenvironment [3–7]. A primary objective of radiomics is to provide predictive imaging biomarkers that, in conjunction with clinical parameters, could improve diagnosis and treatment prognostication, quality of life, and overall survival (OS), aligning with personalized and precision medicine goals.

Despite the substantial volume of NSCLC radiomics research, the translation into clinical practice has been constrained by technical and methodological challenges, resulting in studies with low statistical power and decreased replicability, reproducibility, and generalizability [3, 8–13]. Quality scoring tools and checklists, such as the Radiomics Quality Score (RQS) with 16 items and a maximum point score of 36, and the CheckList for EvaluAtion of Radiomics Research (CLEAR) with 58 items but without point-scoring, have been developed to address these challenges [10, 14]. However, their adoption has been limited, and concerns persist regarding their reliability in uniformly assessing the quality of radiomics research [9, 15].

Our study aims to 1) identify promising radiomics biomarkers in stage III/IV NSCLC treated with radiation in the literature and 2) critically appraise the research pipeline using the recently published CLEAR and longer-existing RQS systems, and merge the wording of both CLEAR and RQS frameworks into a comprehensive checklist (CLEAR-RQS) allowing a comparison between CLEAR-RQS point-scoring against CLEAR and RQS [9, 10]. CLEAR-RQS aims to serve as a valuable resource to radiomics researchers and educators across various disciplines.

## Materials and methods

For this research, IRB approval was not required since it does not include any human subjects or include any identifiable private information.

### Objective 1: PRISMA literature search to identify radiomics studies in stage III/IV NSCLC patients treated with radiotherapy

**Fig. 1:**
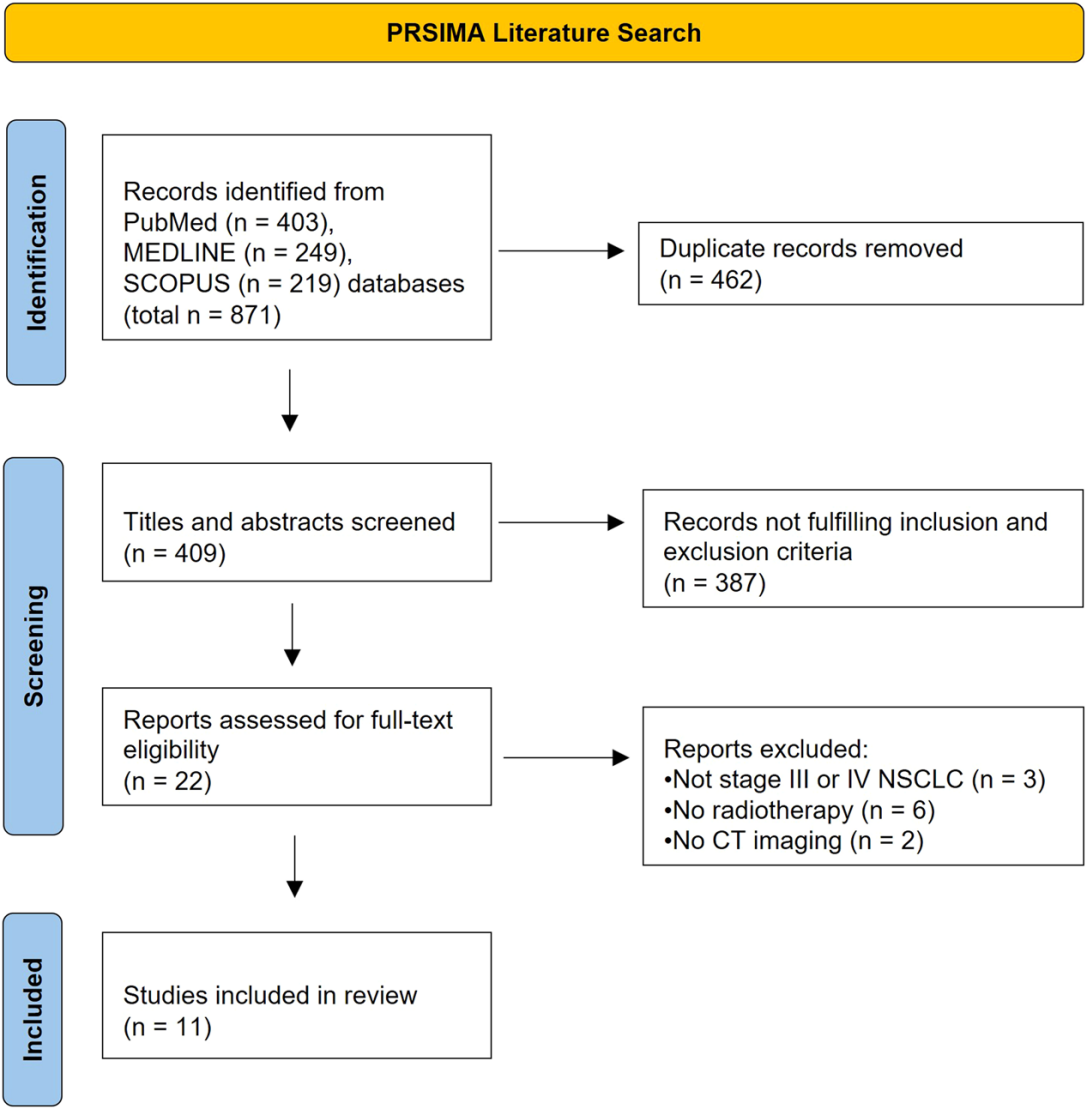
PRISMA flow diagram of PubMed, MEDLINE, and SCOPUS literature search

We conducted a literature search of online databases MEDLINE, PubMed, and SCOPUS from June to August 2023. Search fields comprised of [Stage III NSCLC OR Stage IV NSCLC OR nonsmall cell lung cancer] AND [radiotherapy OR SABR (stereotactic ablative body radiation) OR SBRT (stereotactic body radiation therapy)] AND [CT radiomic OR [quantitative AND imaging] OR [texture AND feature]]. Initial title and abstract analyses were performed by K.T. (3rd-year graduate medical student) with subsequent full-text screening assessment by K.T. and H.S.K. (radiologist with 20 years of general and 16 years of oncological imaging subspecialty knowledge). The final article selection comprised original research in human studies with articles written in the English language on CT radiomics in post-radiotherapy stage III/IV NSCLC (Table 1). Figure 1 shows the PRISMA flow diagram of the literature search.

**Table 1:** PRISMA literature search

Inclusion	Exclusion
• Locally advanced stage III or metastatic stage IV NSCLC as per AJCC • Primary human research • Radiotherapy • Imaging modality: CT • English as primary publication language	• Early stage I or II NSCLC as per AJCC • Not original research (e.g., review, meta-analysis, case study) • Not radiomics research • Not purely radiotherapy (e.g., radio-chemotherapy, radio-immunotherapy)

Applied inclusion and exclusion criteria after an initial literature search (MEDLINE, PubMed, SCOPUS) from June to August 2023

*AJCC* American Joint Committee on Cancer, *NSCLC* nonsmall cell lung cancer

#### Literature data extraction and analysis

Article data extraction included cohort size, radiotherapy/ CT technique, utilized radiomics software, selected RFs, and study endpoints.

Critical appraisal of full-text articles was performed regarding the following research questions: 1) are there commonly selected RFs for treatment response, adverse events, and/ or outcomes in patients undergoing radiotherapy? 2) are there factors within the research study design that would impede reproducibility?

### Objective 2: critical appraisal of selected articles applying CLEAR and RQS frameworks and development of a comprehensive radiomics assessment checklist (CLEAR-RQS)

All articles were assessed by three readers, D.G. (radiologist with 4 years of general radiology experience), K.T., and H.S.K., utilizing the RQS metrics and the CLEAR/ CLEAR-RQS criteria [10, 14]. To facilitate a direct comparison between RQS and CLEAR/ CLEAR-RQS, a point score of 1 for “yes” and of 0 for “no” or “NA” responses was assigned to each CLEAR/ CLEAR-RQS item, resulting in a maximal possible score of 58 for CLEAR and 61 for CLEAR-RQS.

The mean score from all three readers was utilized to compare the RQS, CLEAR, and CLEAR-RQS frameworks. To enable a relative comparison between frameworks, the score of each tool was proportionally converted to a percentage based on its metric (e.g., 100% equated to a CLEAR point score of 58, a CLEAR-RQS score of 61, and an RQS score of 36).

K.T. and H.S.K. systematically compared the wording and interpretation of all 58 CLEAR and 16 RQS items (Table 2). To prevent redundancy, identical and very similar items were merged, retaining the wording of the more specific source framework (CLEAR or RQS). No new wording was introduced to ensure adherence to the respective source framework.

**Table 2:** CLEAR-RQS checklist

Domain Heading	CLEAR item number. Wording	RQS item number. Wording	Comments/Reasoning	CLEAR-RQS item number. Wording
**Title**	1 Relevant title, specifying the radiomic methodology			1 Relevant title, specifying the radiomic methodology
**Abstract**	2 Structured summary with relevant information			2 Structured summary with relevant information
**Keywords**	3 Relevant keywords for radiomics			3 Relevant keywords for radiomics
**Introduction**	4 Scientific or clinical background			4 Scientific or clinical background
	5 Rationale for using a radiomic approach			5 Rationale for using a radiomic approach
**Method: study design**	6 Study objective(s)			6 Study objective(s)
	7 Adherence to guidelines or checklists (e.g., CLEAR checklist)		Optional for quality assurance of radiomics research	6.1 Optional: Adherence to guidelines or checklists (e.g., CLEAR checklist)
	8 Ethical details (e.g., approval, consent, data protection)			7 Ethical details (e.g., approval, consent, data protection)
	9 Sample size calculation			8 Sample size calculation
	10 Study nature (e.g., retrospective, prospective)	11 Prospective study registered in a trial database— provides the highest level of evidence supporting the clinical validity and usefulness of the radiomics biomarker	RQS is more specific about study design	9 Study nature (e.g., retrospective, prospective)
	11 Eligibility criteria			10 Eligibility criteria
	12 Flowchart for technical pipeline			11 Flowchart for technical pipeline
**Method: Data**	13 Data source (e.g., private, public)	1 Image protocol quality—well-documented image protocols (for example, contrast, slice thickness, energy, etc.) and/or usage of public image protocols allow reproducibility/replicability	RQS weighting Both focus on the reporting of the original source and quality of imaging. CLEAR criteria wording was retained as it separated out both data source (public or private) and image acquisition protocols accordingly	12 Image protocol quality: Data source (e.g., private, public)
	14 Imaging protocol (i.e., image acquisition and processing)			13 Imaging protocol quality (i.e., image acquisition and processing)
		3 Phantom study on all scanners—detect inter-scanner differences and vendor-dependent features. Analyze feature robustness to these sources of variability	No clear correspondence to the CLEAR criteria	14 Phantom study on all scanners—detect inter-scanner differences and vendor-dependent features. Analyze feature robustness to these sources of variability
		4 Imaging at multiple time points—collect images of individuals at additional time points. Analyze feature robustness to temporal variabilities (for example, organ movement, organ expansion/shrinkage)	Not clear correspondence to CLEAR criteria—some overlap to imaging protocol (16)	15 Imaging at multiple time points—collect images of individuals at additional time points. Analyze feature robustness to temporal variabilities (for example, organ movement, organ expansion/shrinkage)
	15 Data overlap			16 Data overlap
	16 Data split methodology			17 Data split methodology
	17 Definition of nonradiomic predictor variables		Optional: depends on study design	18 Optional: Definition of nonradiomic predictor variables- depends on study design
	18 Definition of the reference standard (i.e., outcome variable)			19 Definition of the reference standard (i.e., outcome variable)
**Method: Segmentation**	19 Segmentation strategy	2 Multiple segmentations possible actions are segmentation by different physicians/algorithms/software, perturbing segmentations by (random) noise, and segmentation at different breathing cycles. Analyze feature robustness to segmentation variabilities	RQS weighting Both focus on the reporting segmentation and harmonization strategies of imaging data to ensure reproducibility. The wording of CLEAR was retained as it was deemed more specific in describing key steps in image pre-processing and data harmonization protocols.	20 Segmentation strategy
	20 Details of operators performing segmentation			21 Details of operators performing segmentation
	21 Image pre-processing details			22 Image pre-processing details
**Pre-processing**	22 Resampling method and its parameters			23 Resampling method and its parameters
	23 Discretization method and its parameters			24 Discretization method and its parameters
	24 Image types (e.g., original, filtered, transformed)			25 Image types (e.g., original, filtered, transformed)
**Feature extraction**	25 Feature extraction method	5 Feature reduction or adjustment for multiple testing—decreases the risk of overfitting. Overfitting is inevitable if the number of features exceeds the number of samples. Consider feature robustness when selecting features	RQS weighting Both focus on reporting feature selection and reduction methods. The wording of CLEAR was retained as it was deemed more detailed in this instance, focusing in individual steps for feature extraction and data preparation	26 Feature extraction method
	26 Feature classes			27 Feature classes
	27 Number of features			28 Number of features
	28 Default configuration statement for remaining parameters			29 Default configuration statement for remaining parameters
**Data preparation**	29 Handling of missing data			30 Handling of missing data
	30 Details of class imbalance			31 Details of class imbalance
	31 Details of segmentation reliability analysis			32 Details of segmentation reliability analysis
	32 Feature scaling details (e.g., normalization, standardization)			33 Feature scaling details (e.g., normalization, standardization)
	33 Dimension reduction details			34 Dimension reduction details
**Modelling**	34 Algorithm details			35 Algorithm details
	35 Training and tuning details	12 Validation—the validation is performed without retraining and without adaptation of the cut-off value, provides crucial information with regard to credible clinical performance	RQS weighting Both focus on reporting processes regarding testing of the developed model, including resampling methods (random train-test split, cross-validation, bootstrap validation etc.) The wording of CLEAR was retained	36 Training and tuning details
	36 Handling of confounders			37 Handling of confounders
	37 Model selection strategy			38 Model selection strategy
**Evaluation**	38 Testing technique (e.g., internal, external)			39 Testing technique (e.g., internal, external)
	39 Performance metrics and rationale for choosing			40 Performance metrics and rationale for choosing
	40 Uncertainty evaluation and measures (e.g., confidence intervals)	8 Cut-off analyzes—determine risk groups by either the median, a previously published cut-off or report a continuous risk variable. Reduces the risk of reporting overly optimistic results	RQS weighting Both focus on statistical performance comparison The wording of CLEAR was retained	41 Uncertainty evaluation and measures (e.g., confidence intervals)
	41 Statistical performance comparison (e.g., DeLong’s test)			42 Statistical performance comparison (e.g., DeLong’s test)
	42 Comparison with non-radiomic and combined methods	6 Multivariable analysis with nonradiomics features (for example, EGFR mutation)—is expected to provide a more holistic model. Permits correlating/inferencing between radiomics and non-radiomics features	Both report how radiomic features can be combined with non-radiomic features. The wording of CLEAR and RQS was merged	43 Comparison with non-radiomic and combined methods: Multivariable analysis with non-radiomics features
	43 Interpretability and explainability methods			44 Interpretability and explainability methods
**Results**	44 Baseline demographic and clinical characteristics			45 Baseline demographic and clinical characteristics
	45 Flowchart for eligibility criteria			46 Flowchart for eligibility criteria
	46 Feature statistics (e.g., reproducibility, feature selection)			47 Feature statistics (e.g., reproducibility, feature selection)
	47 Model performance evaluation	9 Discrimination statistics—report discrimination statistics (for example, C-statistic, ROC curve, AUC) and their statistical significance (for example, *p* values, confidence intervals). One can also apply resampling method (for example, bootstrapping, cross-validation)	RQS weighting Both focus on reporting statistical methods of assessing model performance. The wording of RQS was retained as it was deemed more specific in this instance as it detailed specific statistical tests that are to be performed	48 Model performance evaluation: Discrimination statistics
		10 Calibration statistics - report calibration statistics (for example, Calibration-in-the-large/slope, calibration plots) and their statistical significance (for example, *P* values, confidence intervals). One can also apply resampling method (for example, bootstrapping, cross-validation)		49 Model performance evaluation: Calibration statistics
	48 Comparison with non-radiomic and combined approaches	13 Comparison to ‘gold standard’—assess the extent to which the model agrees with/is superior to the current ‘gold standard’ method (for example, TNM-staging for survival prediction). This comparison shows the added value of radiomics	RQS weighting Both focus on reporting how radiomics model compared to non-radiomic approaches The wording of RQS was retained as it was deemed more specific in this instance	50 Comparison with non-radiomic and combined approaches: Comparison to ‘gold standard'
		7 Detect and discuss biological correlates—demonstration of phenotypic differences (possibly associated with underlying gene–protein expression patterns) deepens understanding of radiomics and biology		51 Comparison with non-radiomic and combined approaches: Detect and discuss biological correlates
**Discussion**	49 Overview of important findings			52 Overview of important findings
	50 Previous works with differences from the current study	14 Potential clinical utility—report on the current and potential application of the model in a clinical setting (for example, decision curve analysis).	Both focus on reporting of potential clinical utility and real-world application of radiomic models. The wording of CLEAR was merged with RQS	53 Practical implications: Potential clinical utility
	51 Practical implications	15 Cost-effectiveness analysis—report on the cost-effectiveness of the clinical application (for example, QALYs generated)	RQS weighting	54 Previous works with differences from the current study
	52 Strengths and limitations (e.g., bias and generalizability issues)			55 Strengths and limitations
**Data availability**	53 Optional: Sharing images along with segmentation data	16 Open science and data—make code and data publicly available. Open science facilitates knowledge transfer and reproducibility of the study	Both focus on open science and sharing data files The wording of CLEAR was retained as it was deemed more specific in this instance	56 Optional: Sharing images along with segmentation data
	54 Sharing radiomic feature data			57 Sharing radiomic feature data
**Code availability**	55 Sharing pre-processing scripts or settings			58 Sharing pre-processing scripts or settings
	56 Sharing source code for modelling			59 Sharing source code for modelling
**Model availability**	57 Sharing final model files			60 Sharing final model files
	58 Optional: Sharing a ready-to-use system			61 Optional: Sharing a ready-to-use system

Listing of CLEAR domains, CLEAR and RQS item numbers/wording and reasoning for merge

## Results

### Objective 1: PRISMA literature search

Figure 1 demonstrates the PRISMA diagram, which outlines the literature search. In total, 871 articles were found (PubMed *n* = 403, MEDLINE *n* = 249, SCOPUS *n* = 219). After the exclusion of 462 duplicates, 409 article abstracts were screened. This resulted in 22 identified articles that underwent full-text assessment, of which a further 11 were excluded based on inclusion and exclusion criteria (Table 1). Finally, 11 articles were included in the systematic review (Supplemental Table S1).

#### Cohort specifics

Retrospective patient cohort sizes ranged from 10 to 337 (median = 91, mean =114), with 7 studies comprising smaller cohort sizes of less than 100 [11, 16–21]. All studies except for 2 analyzed single-center patient cohorts [11, 22].

#### Study endpoints of selected radiomic features

Study endpoints varied with selected RFs relating to OS in three studies [17, 23, 24] and to treatment response in two studies [19, 25]. Three studies analyzed both OS and progression-free survival [11, 21, 22], and two studies examined the treatment-related complication of radiation pneumonitis [18, 20]. One study measured RF changes in the NSCLC tumor before and during radiotherapy without association with any clinical endpoint [16].

#### Radiotherapy regimen

Applied radiotherapy methods varied, with intensity-modulated radiotherapy (IMRT) utilized in three studies, IMRT or stereotactic body radiotherapy in one study, volumetric modulated arc therapy (VMAT) used in two studies [16, 18, 21, 23, 25, 26]. One study employed stereotactic ablative radiotherapy (SBRT) in a subset of its patient cohort [23]. Four studies did not mention specific radiotherapy delivery methods [17, 19, 20, 22].

#### CT imaging protocol

CT vendor/ scanner type and scanning technique varied or were not disclosed in multiple aspects.

Regarding CT vendor and scanner models, 6 out of 11 articles mentioned the scanner type model, and out of these 6, 5 used a single CT scanner model.

Two studies used noncontrast cone beam CT images [17, 22].

Three studies used contrast-enhanced CT images [11, 21, 24], and the remaining 6 studies did not mention specific contrast phases [16, 18–20, 23, 25].

Three studies specified the respiratory cycle timepoint of image acquisition, with 2 at free breathing cycles [18, 20] and 1 at the end-expiratory phase [21].

Three studies did not specify the CT slice thickness [19, 24, 25], and 4 studies reported a CT slice thickness of 2.5 mm [11, 16, 20, 22].

One study each analyzed 1 or 2 mm [23], 1 or 3 mm [17], 2.5 or 3.0 mm [21], and 5 mm [18] CT slice thicknesses, respectively.

#### Radiomic feature extraction

RF extraction software was highly variable among the studies. Eight studies extracted features utilizing common software tools (1 AnalysisKit [23], 2 PyRadiomics [18, 20], 2 IBEX [16, 17], 3 MATLAB [11, 19, 22], 1 LIFEx [25]). One study employed an in-house software to extract radiomic features [21], and 1 study did not disclose the utilized software [5].

#### Radiomic feature selection

Full-text analysis scoring revealed a lack of similarities to identify common RFs given the variability of study endpoints (e.g., treatment response, OS, radiotherapy-related pneumonitis), along with differing data sets. Grey-Level Co-occurrence Matrix (GLCM)[11, 16-20; 22, 24, 25], first-order RFs intensity [16, 17, 20, 22, 23] and shape [17, 20, 22, 23], and higher order RF Grey-Level Size Zone Matrix (GLSZM) [18, 23, 25], were among the selected RFs described.

#### Model building

Model or nomogram building with non-RF parameters was described in 8 out of 11 studies [11, 18, 19, 21–25]. Available model/ nomogram performance varied, with three studies demonstrating borderline significant *p* values of 0.048, 0.049, and 0.046, respectively [11, 21, 23]. Most common utilized clinicopathological parameter for model building was smoking [18, 19, 21, 25], T- and N-stage [19, 21, 22, 25], with each factor observed in four studies, followed by tumor histology incorporated in three studies [19, 21, 22].

Supplemental Table S1 describes the articles’ detailed data extraction.

### Objective 2: applying CLEAR and RQS point-scoring to selected articles (*n* = 11) and development of a comprehensive radiomics assessment checklist (CLEAR-RQS)

#### CLEAR metrics

The median CLEAR point score was 32.33 (55.74%, range: 25.33–48 [47.7–82.75%]). Across all three readers, all studies fulfilled the “manuscript preparation” CLEAR criteria of providing a title, abstract, keywords, introduction, and discussion. All articles failed to report details regarding the items “sample size calculation” and “flowchart for eligibility criteria”, and the entire domain of “open science.”

Table 3 summarizes the 44 items in detail where two or all three readers identified missing data pertaining to the respective CLEAR item.

**Table 3:** Missing data on CLEAR framework




CLEAR items metric where two (light grey) or three readers (dark grey) identified missing data scored 0 on the respective articles (gray fields). Articles are listed in alphabetical order

#### RQS metrics

The median RQS point-score was 6.33 (17.59%) with a range of 0-16 points (0–44.44%) out of a maximal possible 36-point-score. Many criteria scored 0 or below by all readers as illustrated in Table 4, e.g., no study contained “phantom calibrations”, were “prospective studies registered with a database”, or performed a “cost-effective analysis”.

**Table 4:** Missing data on RQS framework

RQS item metric where two (light grey) or three readers (dark grey) scored 0 or less on the respective articles. Articles are listed in alphabetical order

#### Comparing CLEAR and RQS point distribution

Table 5 demonstrates the point distribution for papers evaluated using the CLEAR and RQS criteria. Ranking differed for the top 3 articles when using the CLEAR versus RQS systems, for example, Chen et al [23] ranked 1st on the RQS but 4th according to CLEAR metrics, whereas Van Timmeren et al [22] ranked 1st on the CLEAR but 2nd according to the RQS framework.

**Table 5:** RQS and CLEAR scores and rankings

RQS and CLEAR scores of 11 articles on radiomics quality in stage III/IV NSCLC treated with radiotherapy (in alphabetical order). The top 3 ranks according to RQS and CLEAR scoring have been highlighted by a gray cell background color with the top 1 rank in bold

* Equal 10th

**Fig. 2:**
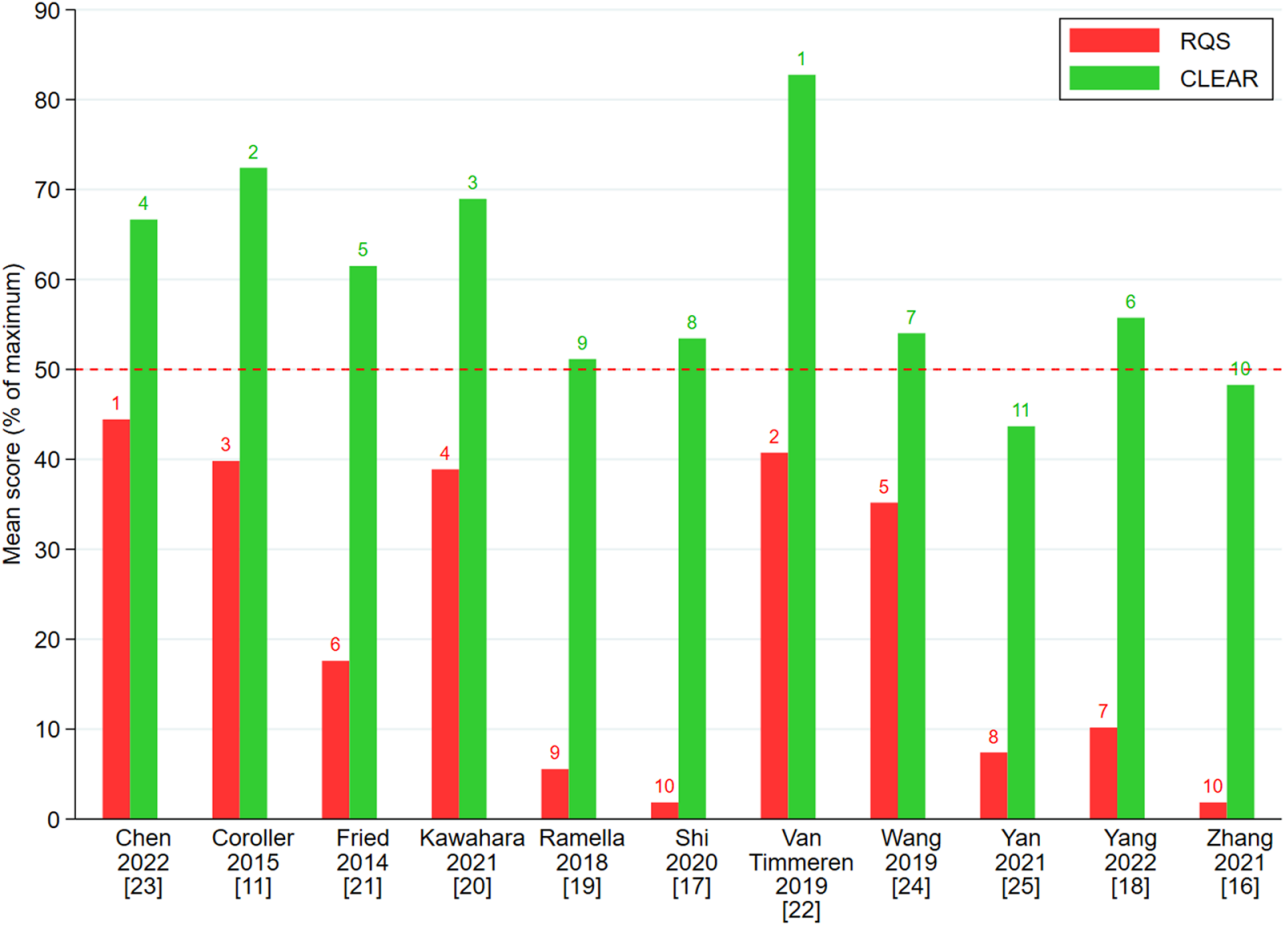
RQS and CLEAR percentage score distributions of assessed radiomics articles in post-radiotherapy stage III/IV NSCLC (*n* = 11). Red bars representing the RQS, and green bars representing the CLEAR, frameworks. Numbers on top of the bars represent the RQS and CLEAR rank, respectively. The horizontal red bar delineates 50% percent highlighting that no RQS score was above 50%. Articles are listed in alphabetical order

Figure 2 shows the score point values and respective ranking of appraised articles according to the CLEAR and RQS metrics.

### Amalgamation of CLEAR and RQS items into a comprehensive assessment checklist (CLEAR-RQS) and comparing CLEAR-RQS with CLEAR and RQS

The 58 CLEAR and 16 RQS items’ wording was compared and identical or similar, resulted in the merging of items and the development of a 61-item CLEAR-RQS checklist (Table 2).

When applying the newly developed CLEAR-RQS checklist, the scoring percentage of each article was between its CLEAR and RQS score, with CLEAR-RQS adhering closer to the CLEAR checklist (Supplemental Fig. S1). This is easily explained, given that the CLEAR-RQS checklist contains 61 items, which is much more aligned with the 58-item containing CLEAR checklist compared to the RQS framework which only contains 16 items.

## Discussion

This systematic literature review on radiomic features in post-radiotherapy stage III/IV NSCLC patients yielded 11 retrospective studies, exhibiting substantial variations in their study design, rendering them incomparable, and failing to identify an RF suitable for clinical translation. Moreover, there was low reporting quality when applying both the CLEAR and RQS frameworks, consistent with findings from other radiomics data reviews and meta-analyses [8, 15, 27, 28]. Merging the CLEAR and RQS frameworks into a comprehensive CLEAR-RQS checklist aimed to provide a comprehensive yet detailed guide for designing and critically appraising published research to the radiomics research community.

### Limitations in radiomics study design

This review revealed several shortcomings in research design, potentially diminishing the generalizability and reproducibility of identified RFs.

*The heterogeneity* of study cohorts and relatively small sample sizes may limit comparability. Notably, two studies featured small sample sizes (*n* = 10, *n* = 23), rendering validation nearly unfeasible [16, 17].

*Data harmonization*, particularly image acquisition and reconstruction settings (referred to as “pre-processing” by CLEAR and RQS), emerged as a key requirement in radiomics research [29, 30]. Three studies did not disclose whether CT slice thickness harmonization was performed [19, 24, 25]. Body habitus, scanner models, and demographic parameters may influence radiomic analysis, necessitating their specifications for future validation [30]. This may require further data postprocessing to ensure reproducibility [29]. Two studies [17, 22] used cone-beam CT (CBCT) images, introducing challenges related to radiomic region-of-interest delineation caused by scattered radiation artifacts [31, 32]. Only three studies detailed the use of free breathing CT images [17, 18, 20], with the remaining studies neglecting to specify the CT acquisition breathing cycle point [11, 16, 19, 21–25]. Free-breathing studies introduce image blurring due to movement artefacts, acknowledged to impact radiomics analysis [33]. Consequently, RF extraction from inherently inconsistent or highly variable CT scanning protocols may compromise result interpretation and reproducibility.

Seven studies omitted reporting of image *pre-processing resampling techniques* and associated parameters [11, 16–19, 24, 25]. Eight studies failed to describe *discretization methods* [11, 16–20, 24, 25]. Image resampling, particularly downsampling and interpolating images in a manner that preserves spatial detail while avoiding overfitting, is critical for data harmonization. Shafiq-ul-Hassan et al demonstrated that resampling could reduce feature variability, therefore enhancing RF robustness [34].

Only 2 studies reported details of *feature extraction segmentation of reliability analysis* [11, 21]. Description of this step is important, as manual or semi-automated segmentation methods may introduce intra- and inter-observer variability, impacting reproducibility [35].

Certain categories of RFs, including first-order (intensity, shape) and higher-order (GLCM (Grey-Level Co-Occurrence Matrix), GLSZM (Grey-Level Size Zone Matrix)) groups, were more commonly investigated [11, 16–20, 22, 24, 25].

### CLEAR and RQS metrics to assess the quality of radiomics research reporting

#### Item weighting

Assessing study quality depends on robust research design and comprehensive reporting of methodology, statistical parameters, and results. Both CLEAR criteria and RQS scores indicated suboptimal reporting quality, with variations in study rankings. No study fully met all CLEAR items, and RQS scores ranged from 0 to +16 points, less than 50% of the maximum achievable +36 points. Our analysis suggests that these assessment tools offer complementary critiques for identifying methodological challenges hindering the reproducibility and clinical application of radiomic results.

The CLEAR checklist offers a general guideline covering all aspects of the radiomics workflow, while the RQS framework comprises 16 criteria with varying weighted point scores. Certain domains, such as “prospective validation in an appropriate trial” (0 or +7 points) and “validation cohorts” (-5, +2, +3, +4, +5 points), are assigned more points compared to others. These items contributed most to top-scoring papers on RQS, which did not align with their CLEAR ranking. For instance, the RQS item “validation” negatively impacted the scores of Yang et al (3.67 points) [18], Shi et al (0.67 points) [17], and Zhang et al (0.67 points) [16], ranking them 7th, 10th, and 11th out of 11 articles, respectively. Such large point score disparities were not observed with CLEAR criteria, as exemplified by the comparison of Wang et al and Fried et al [21, 24]. With RQS, Wang et al ranked 5th (12.67 points) while Fried et al ranked 6th (6.33 points), whereas in the CLEAR metric, the point scoring disparity was less evident, and with Wang et al ranking lower (rank 7, 31.33 points) than Fried et al (rank 5, 35.67 points [21, 24].

A recently published quality scoring tool for radiomics research, METRICS (METhodological RadiomICs Score), has been developed by an international panel and has been endorsed by the European Society of Medical Imaging Informatics (EUSoMII). METRICS contains weighted items carefully selected and discussed via a modified Delphi process to ensure a balanced consensus among panelists [36]. This new point-scoring framework aims to facilitate critical appraisal of a broad range of radiomics research, from the manual data labeling and extraction to deep learning artificial intelligence (AI) pipelines.

#### Inter-rater variability

D’Antonoli et al’s study revealed that the RQS metric is susceptible to inter-rater biases, as its domains can be construed differently depending on raters’ backgrounds [9]. This corresponds to our findings, as our three raters – a graduate medical student, a junior radiologist, and a senior radiologist – exhibited minor discrepancies in RQS scores, which were reconciled through consensus. This variability aligns with prior research indicating low RQS scores and poor inter-rater reliability [9, 27, 28].

### Creating a comprehensive CLEAR-RQS checklist to aid future education and research

Efforts aim to develop a robust tool for assessing radiomics research quality, with a focus on machine learning and other AI models [37–39]. The RQS and CLEAR frameworks specifically address radiomics methodology [10, 14], which has garnered attention from the Society of Nuclear Medicine and Molecular Imaging, the European Association of Nuclear Medicine [39], and the Scientific Editorial Board of European Radiology [40].

The herein presented CLEAR-RQS checklist, developed by an international research group from two academic tertiary institutions, aims to comprehensively evaluate radiomics methodologies, without sacrificing specificity. It integrates standards from both CLEAR and RQS tools, preserving their detailed wording catering to radiomics researchers, while also serving educational purposes across various disciplines. The application of a point-scoring system to the CLEAR-RQS checklist should be avoided, given the intricate complexities inherent in real-world research scenarios, which may not be granular enough to adequately capture the nuanced quality of the assessed research investigations.

In conclusion, stage III/IV NSCLC radiomics research suffers from suboptimal reporting quality, hindering the discovery of validated predictive RFs. Technical challenges and lack of access to source images and model files impede reproducibility. Thorough validation and open access to data and code are essential to increase transparency and raise reporting standards [41, 42]. Adoption of the CLEAR-RQS checklist could accelerate the translation of radiomics research into clinical practice. Furthermore, sustained multi-disciplinary collaboration for continuous assessment and improvement in this rapidly evolving field is required to ultimately benefit patient outcomes in personalized medicine.

## Supplementary information

Electronic Supplementary Material

## Abbreviations

CLEAR: Checklist for evaluAtion of radiomics research
GLCM: Grey-level co-occurrence matrix
GLSZM: Gray-level size zone matrix
IMRT: Intensity-modulated radiotherapy
NSCLC: Non-small cell lung cancer
OS: Overall survival
RF: Radiomic feature
RQS: Radiomics quality score
SBRT: Stereotactic body radiation therapy
