# Supplementary material for: Post-radiotherapy stage III/IV non-small cell lung cancer radiomics research: a systematic review and comparison of CLEAR and RQS frameworks
Source: Eur Radiol. 2024 Apr 16;34(10):6527–43. doi: 10.1007/s00330-024-10736-1 (PMC11399214; doi:10.1007/s00330-024-10736-1)
Supplement: Supplementary file 1 — Electronic Supplementary Material [file 330_2024_10736_MOESM1_ESM.pdf]

Post-radiotherapy Stage III/IV Non-Small Cell Lung Cancer Radiomics Research: A Systematic Review and Comparison of CLEAR and RQS frameworks

ELECTRONIC SUPPLEMENTARY MATERIAL

Eur Radiol (2024) Tran K, Ginzburg D, Hong W, Attenberger U, Ko HS.

Supplemental Table S1: Characteristics and radiomic features of included full text articles on radiomics in stage III/ IV NSCLC post radiotherapy (alphabetical order of authors).

Authors (year) [reference]	Publishing journal	Study endpoint(s)	Patient cohort size (sub cohorts)<br><br>Number of centers	Radiotherapy type<br><br>Dose<br><br>Fraction	CT scanner<br><br>CT technique<br><br>Contrast phase<br><br>Breathing cycle	CT slice thickness	Radiomics software	Radiomic features<br><br>Model building non RF parameters (Model)	General and further comments on radiomics study reporting
Chen et al (2022) [23]	Technology in Cancer Research and Treatment	OS<br><br>Radiotherapy sensitive versus non-radiotherapy sensitive	104<br>(73 training, 31 validation)<br><br>Single center	IMRT<br>60-66y<br>30-33 fractions<br>or<br>SBRT<br>45-54Gy<br>5-10 fractions	Siemens Somatom definition<br>Or<br>Philips Brilliance<br><br>MDCT<br><br>Contrast phase ND<br><br>Breathing cycle ND	1 or 2mm	AnalysisKit	First order intensity, shape<br>Higher order GLDM, GLSZM<br><br>Model: Concurrent chemoradiation in differing radiosensitive vs non-radiosensitive p=0.048	Heterogenous radiation regimen<br><br>Some patients undergoing concurrent chemotherapy<br><br>CT technique<br><br>Significance for selection of features defined as p< 0.15
Coroller et al (2015) [11]	Radiotherapy and Oncology	OS<br><br>Time to distant metastasis	98<br>(84 validation)<br><br>Single center	Various<br>Fractions ND	GE LightSpeed<br><br>MDCT<br><br>CE<br><br>Breathing cycle ND	2.5mm	MATLAB	**For OS:**<br>First order skewness, entropy, kurtosis<br>Model: ND<br><br>**For time to distant metastasis:**<br>First order entropy, skewness<br>Higher order GLCM<br><br>Model: Tumor grade and overall stage<br>P=.049	Heterogenous radiation regimen
Fried et al (2014) [21]	International Journal of Radiation Oncology-Biology Physics	OS<br><br>Locoregional control<br><br>Time to distant metastasis	91<br><br>Single center	Various<br>1.8-2Gy/ fraction	NA<br><br>MDCT<br><br>CE<br>and<br>4D NC<br>(Avg CT and T50 CT)	2.5 or 3 mm<br><br>(Most common 2.5mm)	In house software	**For CE CT**<br>First order size, kurtosis<br>NGTDM, COM<br><br>**For AVG CT**<br>First order uniformity<br><br>**For T50 CT**	Heterogenous radiation regimen<br><br>CT technique<br><br>Software for feature extraction ND

					For CE Breathing cycle ND<br><br>For 4D NC, end expiratory phase			First order size, uniformity<br><br>Model: T stage, N stage, Overall stage, gender, histology, Charlson Comorbidity Index, ECOG, Karnofsky, smoking, estimated pack years, gross tumor volume<br>for OS, p=0.046<br>for locoregional recurrence, p=0.01<br>for time to distant metastasis, p=0.005	Feature selection ND<br><br>Heterogeneous image segmentation process
Kawahara et al (2021) [20]	Nature: Scientific Reports	Radiation pneumonitis	77	3D-CRT and VMAT<br><br>60-74Gy Fractions ND	GE LightSpeed<br><br>MDCT<br><br>Contrast phase ND<br><br>Free Breathing	2.5mm	PyRadiomics	First order intensity, shape, skewness<br>Higher order GLCM, GLDM<br><br>Model: none	Heterogenous radiation regimen
Ramella et al (2018) [19]	PLoS One	Tumor volume change	91	45- 75 Gy<br>25-40 fractions	NA<br><br>MDCT<br><br>Contrast phase ND<br><br>Breathing cycle ND	ND	MATLAB	First order LBP<br>Higher order GLCM<br><br>Model: N stage, histology, sex, EGFR mutation, smoking, ALK-mutation, T stage, age<br>Model AUC = 0.820<br>Vs<br>Model without RFs AUC = 0.776	Heterogenous radiation regime<br><br>Some patients undergoing concurrent chemotherapy<br><br><br>CT technique
Shi et al (2020) [17]	Physics in Medicine and Biology	OS	23	>58Gy<br>2Gy/ fraction	NA<br><br>Cone Beam CT<br><br>NC<br><br>Free breathing	1 and 3mm	IBEX	First order intensity, shape<br>Higher order GLCM<br><br>Model: none	Small cohort<br><br>Chemoradiation<br><br>Heterogenous cohort (various CBCT timing)
Van Timmeren et al (2019) [22]	European Journal of Radiology	OS<br><br>Locoregional Recurrence	337<br>(141 training, 3 validation sets: n = 94, 61, 41)	Various<br>>45Gy<br><br>Fractions ND	NA<br><br>Cone Beam CT<br><br>NC<br><br>Free breathing	2.5mm	MATLAB	**For OS and locoregional recurrence**<br>First order intensity<br>Higher order GLCM<br>Vs<br>Model: TNM stage, histology only applicable to 2 of 3 validation sets.<br>C-index = 0.675	Heterogenous radiation regimen<br><br>Some patients undergoing concurrent chemotherapy
Wang et al (2019) [24]	European Journal of Radiology	OS	118	Various	Siemens SOMATOM Definition AS<br><br>MDCT	ND	ND	Higher order GLCM<br><br>Model: age, node metastasis status, hematological blood parameters	Heterogenous radiation regimen

					CE<br><br>Breathing cycle ND			C-index of training =0.792<br>C-index of cross-validation=0.743<br><br>RF alone model C-index=0.699<br>Age, node metastasis model C-index=0.618<br>Hematological blood parameter model C-index=0.653	Data harmonisation ND
**Yan and Wang (2021) [25]**	Science Progress	Tumor volume change	223 (192 training, 31 validation)	IMRT And 3D- CRT 60Gy Fractions ND	NA<br><br>MDCT<br><br>Contrast phase ND<br><br>Breathing cycle ND	ND	LIFEx	Higher order GLCM, GLRLM, GLSZM<br><br>Model: TNM stage, other lung disease, smoking<br>Nomogram: ROC=0.889 for true positive predicting tumor volume shrinkage<br>ROC=.886 for true negative predicting tumor volume non-shrinkage	Heterogenous radiation regimen<br><br>CT technique<br><br>RF selection details ND
**Yang et al (2022) [18]**	BMC Pulmonary Medicine	Radiation pneumonitis	91	VMAT 50–66 Gy 1.8–2 Gy/fraction	Philips Brilliance Big Bore<br><br>MDCT<br><br>Contrast phase ND<br><br>Free breathing	5mm	PyRadiomics	Higher order GLCM, GLSZM, NGTDM<br><br>Model: smoking correlating with lower radiation pneumonitis (nomogram)	CT technique
**Zhang et al (2021) [16]**	European Journal of Radiology	RF change over time	10	VMAT 66–74 Gy Fractions ND	Philips Brilliance<br><br>MDCT<br><br>Contrast phase ND<br><br>Breathing cycle ND	2.5mm	IBEX	First order intensity<br>Higher order GLCM, GLRLM<br><br>Model: none	Very small cohort<br><br>No predictive RF value

Abbreviations (alphabetical except for column “Radiomic features”): OS: Overall Survival, RF: Radiomic Feature, IMRT: Intensity-Modulated Radiotherapy, 3D-CRT: 3-Dimensional Conformal Radiotherapy, SBRT: Stereotactic Body Radiotherapy, VMAT: Volumetric Modulated Arc Therapy, CBCT: Cone-Beam CT

Abbreviation for column “Radiomic Features” (alphabetical): COM: Co-occurrence Matrix, GLCM: Grey-Level Co-occurrence Matrix, GLDM: Grey-Level Dependence Matrix, GLRLM: Grey-Level Run Length Matrix, GLSZM: Grey-Level Size Zone Matrix, LBP: Local Binary Pattern, NGTDM: Neighboring Grey-Tone Difference Matrix

Supplemental Figure S1: RQS, CLEAR, and CLEAR-RQS percentage score distributions of assessed radiomics articles in post radiotherapy stage III/IV NSCLC (n=11). Red bars representing the RQS, green bars representing the CLEAR, blue bars representing the CLEAR-RQS frameworks. Numbers on top of the bars represent the RQS and CLEAR rank, respectively. The horizontal red bar delineates 50% percent highlighting that no RQS score was above 50%. Articles are listed in alphabetical order.

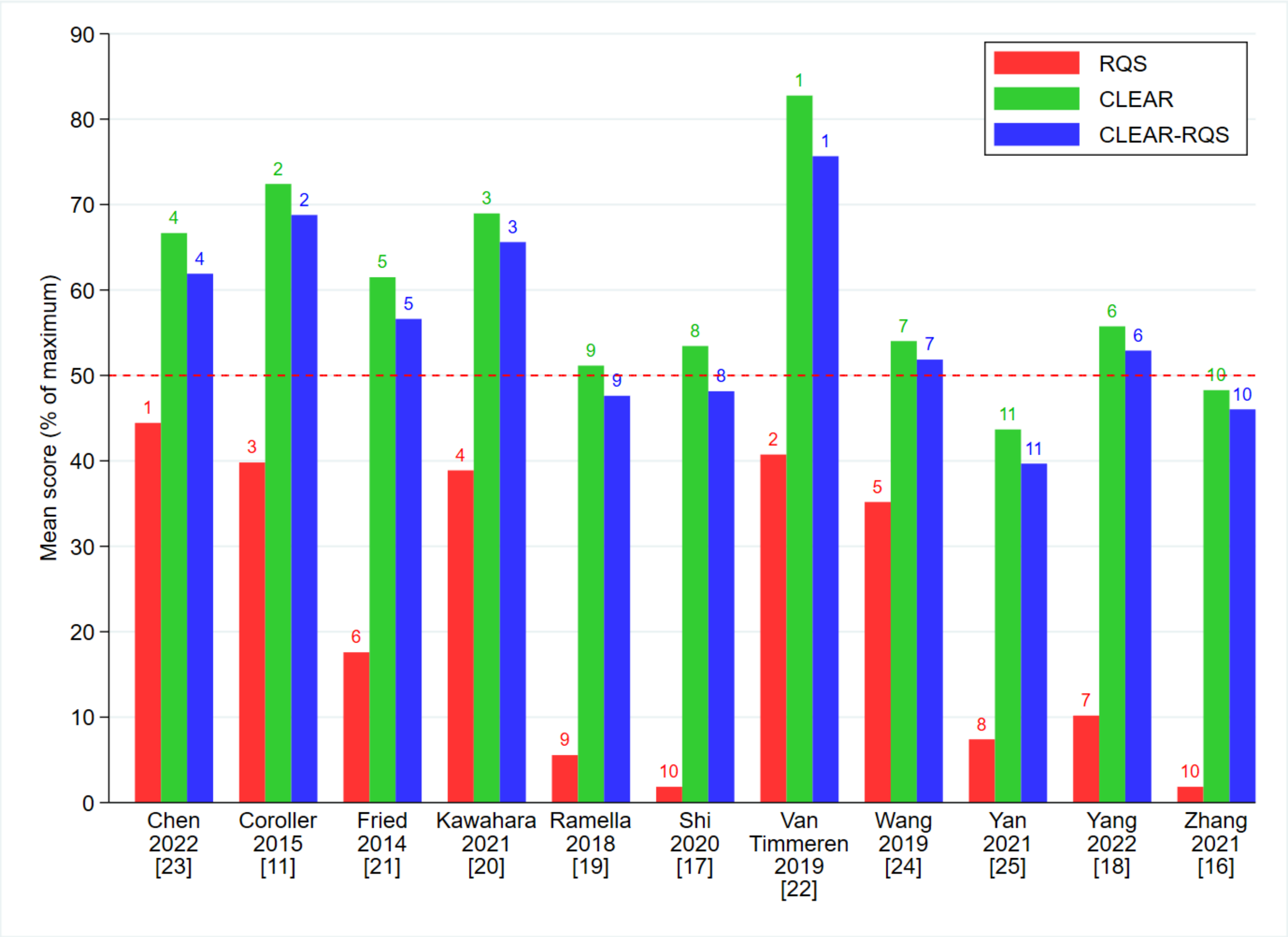

Eur Radiol (2024) Tran K, Ginzburg D, Hong W, Attenberger U, Ko HS.
